# A novel approach to fabricate silk nanofibers containing hydroxyapatite nanoparticles using a three-way stopcock connector

**DOI:** 10.1186/1556-276X-8-303

**Published:** 2013-07-01

**Authors:** Faheem A Sheikh, Hyung Woo Ju, Bo Mi Moon, Hyun Jung Park, Jung Ho Kim, Ok Joo Lee, Chan Hum Park

**Affiliations:** 1 Nano-Bio Regenerative Medical Institute, College of Medicine, Hallym University, Chuncheon 200-702, South Korea; 2 Department of Chemistry, University of Texas-Pan American, Edinburg, Texas 78539, USA; 3 Department of Otorhinolaryngology-Head and Neck Surgery, School of Medicine, Hallym University, Chuncheon 200-702, South Korea

**Keywords:** Nanofibers, Scaffolds, Porosity, Silk fibroin, Hydroxyapatite, Electrospinning

## Abstract

Electrospinning technique is commonly used to produce micro- and/or nanofibers, which utilizes electrical forces to produce polymeric fibers with diameters ranging from several micrometers down to few nanometers. Desirably, electrospun materials provide highly porous structure and appropriate pore size for initial cell attachment and proliferation and thereby enable the exchange of nutrients. Composite nanofibers consisting of silk and hydroxyapatite nanoparticles (HAp) (NPs) had been considered as an excellent choice due to their efficient biocompatibility and bone-mimicking properties. To prepare these nanofiber composites, it requires the use of acidic solutions which have serious consequences on the nature of both silk and HAp NPs. It is ideal to create these nanofibers using aqueous solutions in which the physicochemical nature of both materials can be retained. However, to create those nanofibers is often difficult to obtain because of the fact that aqueous solutions of silk and HAp NPs can precipitate before they can be ejected into fibers during the electrospinning process. In this work, we had successfully used a three-way stopcock connector to mix the two different solutions, and very shortly, this solution is ejected out to form nanofibers due to electric fields. Different blend ratios consisting HAp NPs had been electrospun into nanofibers. The physicochemical aspects of fabricated nanofiber had been characterized by different state of techniques like that of FE-SEM, EDS, TEM, TEM-EDS, TGA, FT-IR, and XRD. These characterization techniques revealed that HAp NPs can be easily introduced in silk nanofibers using a stopcock connector, and this method favorably preserves the intact nature of silk fibroin and HAp NPs. Moreover, nanofibers obtained by this strategy were tested for cell toxicity and cell attachment studies using NIH 3 T3 fibroblasts which indicated non-toxic behavior and good attachment of cells upon incubation in the presence of nanofibers.

## Background

Recent advances in tissue-engineering techniques had enabled scientists in fabricating the novel scaffolds with multi-functional properties to overcome the problems faced in the existing one. The 2D and/or 3D scaffolds used for tissue-engineering applications had greatly influenced the present scenario for scaffold construction. Although there are lots of advances made in tissue-engineering, the scientific community is still facing a major challenge to select the perfect strategy and choice of materials while considering the fabrication of scaffolds. The most uniquely used biopolymer made from silk fibroin proteins are obtained from silkworms and had a long history of applications in the human body as sutures. Silk fibroin contains peptides composed of RGD sequences that can promote cell adhesion, migration, and proliferation [1,2]. These attractive properties of silk fibroin are particularly useful for selecting them as a material of choice for tissue-engineering applications [3]. The efficient biocompatibility, minimal inflammatory response to host tissue, relative slow biodegradation rates compared with other materials, and easy availability from sericulture industry make the silk fibroin a desirable candidate for various medical applications [4].

On the other hand, hydroxyapatite (HAp) is a major solid component of the human bone which can be used as a vital implant due to its excellent biocompatibility, bioactivity, non-immunogenicity, non-inflammatory behavior, and osteoconductive nature [5]. However, the loose and particulate nature of HAp seriously hampers its use in any tissue-engineering applications [6]. In order to utilize the HAp for tissue regeneration especially in the form of scaffolds, it must meet most of the desired requirements, such as desirable mechanical support to sustain the pressure surrounding the host tissues and simultaneously should provide high porosity. For this reason, HAp is often blended with other supporting materials to make its practical utility possible. Desirably, a suitable material is selected to blend with HAp for the facilitation of proper cell seeding and diffusion of nutrients for the healthy growth of cells during the initial period of implant which is considered as crucial [7].

Among available methods, to create a suitable scaffold in which these biologically important materials can be incorporated is the electrospinning technique, which had emerged as a versatile technique to convert biologically significant polymers into nanofibers, so as to use them as potential candidate for tissue-engineering [8-12]. The unique characteristics such as very high surface area-to-volume ratio, high porosity, and capability to mimic the extracellular matrix (ECM) present in the human body had created a special attention on nanofibers produced by the electrospinning technique. Due to these features, electrospun nanofibers had been used as potential candidates for many biomedical applications, such as in drug delivery, wound dressing, and scaffolds for tissue engineering [10-12]. This technique can produce micro- or nanofiber of various polymers in the form of non-woven mats which are similar to the structure present in the natural ECM, which is vital for initial cell adhesion, as a biomimicking factor of cells [13-16]. In this connection, many biodegradable synthetic and/or natural polymers have been electrospun into nanofibers to be used as scaffolds for various tissue repair and regeneration such as bone, cartilage, vascular blood, nerve, skin, and bladder [13-17].

The use of electrospinning to fabricate the silk-based nanofibers and HAp nanoparticles (NPs) had been exploited to create 2D scaffolds. For instance, efforts to modify silk fibroin nanofibers to attribute properties of HAp was done by soaking in stimulated body fluid (SBF) by Kim et al., and this similar mineralization approach had been also frequently used by other researchers [18,19]. However, this soaking method by SBF results in superficial attachment of HAp NPs on nanofibers. In order to have HAp NPs with strong bonding with nanofibers, the use of freeze-dried silk crystals and strong chemicals had been adapted to create nanofibers containing HAp NPs [20,21]. However, it is noteworthy to mention that the use of strong chemicals in that case further restricts the biocompatibility aspect of nanofibers. Therefore, an alternative strategy is needed to fabricate the silk fibroin nanofibers having the features of HAp NPs. The use of aqueous silk/HAp blend solutions can be considered as an ideal way to form nanofibers. By doing that, HAp NPs will be strongly fixed to nanofibers, and intact nature of silk/HAp can be preserved without using toxic chemicals. However, due to large functional groups present in silk, HAp NPs can lead to form a bond due to abundant hydroxyl groups present in these biologically important materials and make it difficult to electrospun [22,23].

In this work, for the first time, we presented the use of aqueous regenerated silk fibroin solution blended with HAp NPs using a three-way stopcock connector. In our system, the aqueous silk solution and HAp NPs colloidal suspension combine together at the center of the three-way connector for a short time without giving enough time to precipitate, and this blend solution is immediately ejected out to form nanofibers. Different weight ratios of 10%, 30%, and 50% of HAp NPs were used as blend solution to electrospun nanofibers. The obtained nanofibers were characterized for various psychochemical characterizations, and interaction of these nanofibers with fibroblasts was done to study the cell toxicity and cell attachment of nanofibers incorporated with HAp NPs.

## Methods

### Materials

Silkworm cocoons were obtained from the Rural Development Administration (Suwon, Republic of Korea). Poly(ethylene oxide) (PEO) with an average molecular weight of 200,000 (Sigma-Aldrich, St. Louis, USA) was used as sacrificial polymer to electrospun silk solution and to make HAp/PEO colloid solutions. HAp rod-shaped NPs measuring 30 to 60 nm were obtained from Dae Jung, Siheung, Gyeonggi, Korea. NIH 3 T3 fibroblasts were purchased from ATCC (Manassas, VA, USA.). Dulbecco’s modified Eagle medium (DMEM) supplemented with 10% fatal bovine serum, cocktail of 1% penicillin-streptomycin, Trypsin, were obtained from Welgene, Fresh Media™ (Dalseogu, Daegu, Korea). Trypan Blue Stain 0.4% was obtained from Gibco® (Life Technologies Corporation, Gaithersburg, MD, USA). 3-(4,5-dimethylthiazol-2-yl)-2,5-diphenyltetrazolium bromide (MTT) reagent used to check the cell viability was purchased from Duchefabiochemie, Haarlem, The Netherlands. Dimethyl sulfoxide (DMSO) with high purity grade of 99.9% was acquired from Sigma-Aldrich. Tissue culture flasks and microplates for cell seeding and growth were purchased from BD Falcon™, Winston-Salem, NC, USA and SPL Life Sciences, Pocheon-si, Gyeonggi-do, Korea.

### Characterization

Variable pressure field emission scanning electron microscope (FE-SEM) EVO® LS10 equipped with energy-dispersive X-ray spectroscopy (EDS) obtained from Carl Zeiss SMT., Ltd., Oberkochen, Germany, was used to investigate the morphology and elemental detection of nanofibers. Before viewing, the samples were pasted on a carbon tape and sputter-coated using a thin layer of gold palladium for 120 s for two consecutive cycles at 45 mA with the Ion Sputter 1010, Hitachi, Chiyoda-ku, Japan. After sample coating, the micrographs from each samples were taken at an accelerating voltage of 2 KV and with magnifications of 15 K. The EDS images were captured at an accelerating voltage of 10 KV and with magnifications of 15 K. The average nanofiber diameters were calculated using the software Innerview 2.0, Dong, Bundang Daeduk Plaza, Korea, after measuring 100 diameters per sample from FE-SEM images. Transmission electron microscopy (TEM) was done by JEOL JEM-2200FS operating at 200 KV, JEOL Ltd., Akishima-shi, Japan. The samples for TEM were prepared by dispersing 10 mg of nanofibers in 200 μl of ethanol and subsequently dispersed by bath sonicator using locally supplied ultrasonic cleaner (60 kHz, Shenzhen Codyson Electrical Co., Ltd., Shenzhen, Guangdong, China) for 120 s. After dispersing the nanofibers, 20 μl of dispersion was pipetted out by micropipette and carefully poured on 200 mesh copper grid. The extra solution was removed using Kimwipes supplied by Kimberly-Clark Professional, GA, USA, and the grid was allowed to dry overnight at room temperature. Information about the phases and crystallinity was obtained using PANalytical diffractometer (HR-XRD, X’pert-pro MPD, Almelo, The Netherlands) with Cu, Cr (*λ* = 1.540 A) radiation over Bragg angle ranging from 10° to 60°. To identify the vibrations caused due to functional groups in nanofibers, Fourier transform infrared spectroscopy (FT-IR) analysis was done using BIO-RAD (Cambridge, MA, USA). The samples were directly loaded on ATR window, and spectra were collected using Excaliber Series by averaging 32 scans with the resolution of 4 cm^−1^. The thermal analysis of the synthesized nanofibers was carried out with a thermal analysis system, (TA Instruments, New Castle, DE, USA) by ramping the samples at 10°C/min, and heating was started from 30°C to 700°C. Heating was followed under a continuous nitrogen purge of 100 mL/min, and spectra were collected using Q600 Software (TA Instruments). For checking the cell attachment on nanofibers by FE-SEM, the images were captured with an accelerating voltage of 3 KV with magnifications of 1 K.

### Preparation of aqueous regenerated silk solutions

The aqueous silk solutions to be used for electrospinning were prepared by the following procedure. Firstly, degumming was achieved by cutting *Bombyx mori* cocoons into suitable pieces and were boiled in 0.02 M Na_2_CO_3_ for an hour and subsequently washed with de-ionized water (2 to 3 times) to remove the unbound sericin. Later on, the samples were dried at room temperature for 1 day. After drying, 60 g of degummed silk was dissolved in ternary solvent composed of CaCl_2_/Ethanol/H_2_O (32/26/42, *wt*/*wt*/*wt*) at 98°C for 40 min in round-bottomed flasks. Following this, protein mixture was filtered through miracloth (Calbiochem, San Diego, CA, USA) to remove small aggregates. Furthermore, this solution was dialyzed against deionized water using a dialysis tubing with molecular weight cutoff 12,000 to 14,000 Da (Spectra/Por®, Rancho Dominguez, CA, USA) for 3 days, and water was exchanged once a day. The yielding aqueous silk fibroin solution was calculated to be 8 wt.% (which was determined by weighing the remaining solid weight after drying). Finally, the aqueous silk fibroin solutions were stored in a refrigerator and used within 15 days of time to avoid denaturation and/or precipitation.

### Nature of used HAp NPs

**Figure 1:**
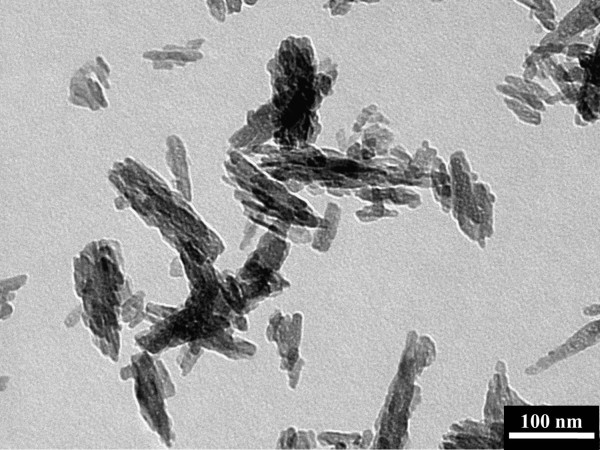
Transmission electron micrograph showing the morphology of used HAp NPs.

Before using the HAp NPs for modifying the nanofibers, the NPs were characterized for shape and size. In this regard, the morphology of obtained HAp NPs was checked by TEM. Figure 1 provides the information about the morphological feature of HAp NPs. From these results, it can be seen that HAp NPs are rod-shaped and are having lengths of 100 to 110 nm and diameters of 20 to 30 nm. These morphology and size provide initial confirmation that they are of appropriate shape and size to fit inside the nanofibers.

### Polymeric solution preparation for electrospinning

For preparing solution to electrospun pristine silk nanofibers, 20 ml of 8 wt.% of aqueous silk solution was removed from the refrigerator. To give appropriate viscosity to this solution, so as to have proper bending instability for fiber formation, 4 ml of previously prepared 30 wt.% PEO solution was added as a ‘sacrificial polymer.’ The resultant blend solutions were loaded in syringes and used for electrospinning. For preparing solutions to fabricate silk fibroin nanofibers containing HAp NPs, a stepwise methodology was adopted. On one hand, silk solution was prepared in the same way as mentioned for the preparation of pristine silk nanofibers and subsequently loaded in syringes. On the other hand, PEO/HAp colloidal solution was prepared by adding 2 g of PEO in 20 ml of 0.001 molar solution of phosphate buffer saline (PBS), and this solution was mixed well to solubilize. To this solution, HAp NPs were added to give the final concentration of 10%, 30%, and 50% HAp with respect to 8% of aqueous silk fibroin solution. After adding HAp NPs in PEO solution, the HAp NPs were agitated using an ultrahigh sonication device. This was achieved using Sonics Vibra-cell model VCX 750, Newtown, CT, USA, operating at 20 kHz with an amplitude of 20%. The ultrasonic agitation was allowed to continue for a period of 1 min. After complete sonication, the samples were viewed as homogeneously dispersed and well stabled without being precipitated at the bottom. Further on, these dispersed HAp/PEO solutions were filled into the syringes to be used for electrospinning.

### Electrospinning process

**Figure 2:**
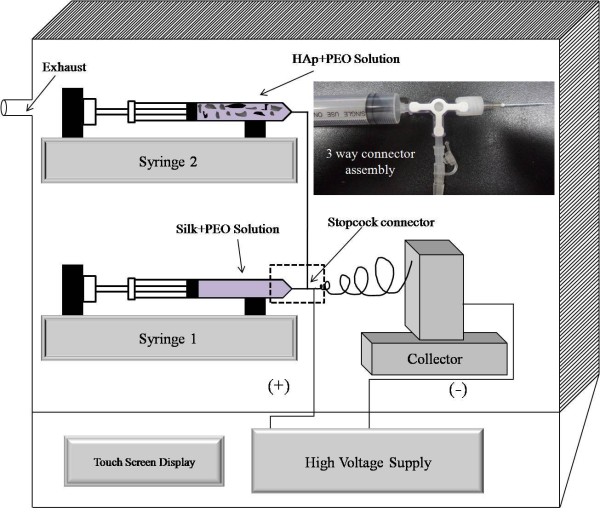
**Schematic presentation of the used electrospinning setup.** The inset image shows the assembly of the stopcock connector used to mix silk/PEO and HAp/PEO colloidal solutions. The inset shows the photograph of the three-way connector used in this study.

The electrospinning of nanofibers was carried out using an electrospinning instrument purchased from eS-robot®, ESR-200R2D, NanoNC, Geumcheon-gu, Seoul, Korea. For fabricating the pristine nanofibers by electrospinning, the silk/PEO solutions were injected using 10 ml disposable plastic syringe fitted with a 22needle gauge (0.7 mm OD × 0.4 mm ID). The syringes were mounted on an adjustable stand, and flow rate of 0.8 mL/min was adjusted using a multi-syringe pump to keep the solution at the tip of the needle without dripping. The high power supply capable of generating +30 kV and −30 kV for positive and negative voltages was used to eject out the nanofibers from the needle tip. A metallic wire originating from the positive electrode (anode) with an applied voltage of +20 kV was connected to the needle tip through alligator clips, and a negative electrode (cathode) with an applied voltage of −1 kV was attached to the flat bed metallic collector [24,25]. The syringes were mounted in the parallel plate geometry at 45° downtilted from the horizontal baseline, and 12 to 15 cm was kept as the working distance (between the needle tip and collector). The as-spun nanofibers were crystallized by incubating the samples in 100%, 70%, 50%, and 0% of ethanol for 10 min each, and samples were frozen and kept for lyophilization overnight. For the electrospinning of nanofibers containing HAp NPs, a three-way stopcock connector was used to mix the silk/PEO and HAp/PEO solutions (Figure 2). As illustrated in Figure 2, from one side, silk/PEO solution was supplied to one of the openings of the stopcock, and from another side, HAp/PEO colloid was supplied to another opening of the stopcock to let solutions blend properly (i.e., silk/PEO + HAp/PEO) and eventually flow towards the needle tip due to the continuous flow rate applied from the syringe pump. All the electrospinning parameters were kept the same as to the electrospun pristine silk nanofibers; the expected flow rate was reduced to 0.4 mL/min, from both syringe pumps, so as to have the final flow rate of 0.8 mL/min (i.e., the flow rate kept for jet formation in case of pristine nanofibers). Furthermore, the nanofibers were treated in the same way for crystallization and freeze drying as aforementioned in case of pristine silk nanofibers.

### Cell viability and cell attachment studies

The frozen ampules of NIH 3 T3 fibroblasts removed from liquid nitrogen tank were incubated at 37°C for 1 to 2 min to form a semisolid suspension. The cells from these ampules were taken out and added with fresh media, centrifuged to get cell debris, and enriched with fresh media allowed to incubate at 37°C for 3 days for the completion of the first subculture. In this study, cells were used after two subcultures to check the cell viability, and cell attachment with renewal of culture media was done after 3 days. The nanofiber samples used for checking cell viability and cell attachment studies were pierced into disk shapes using biopsy punchers (Kasco, Keys Cutaneous Punch, Sialkot, Pakistan) forming 6-mm round disks, giving it an appropriate diameter to fit in a 96 well plate. Each nanofiber disk was sterilized by dipping it in 70% ethanol in 6-well plate for 30 min. The excess of ethanol on nanofibers after sterilization was rinsed by dipping the samples in 10 mL of DMEM. Further on, the nanofiber samples were transferred on 96-well plates in triplicates. A 100 μl of cell suspension containing 25,000 cells/mL was counted using cell counting method, and the cells were carefully seeded over the top of sterilized nanofiber disks in the 96-well plate. The seeded scaffolds were incubated at 37°C for 30 min to allow cell adhesion. Following this, 100 μl of fresh medium was added in each well, and the plates were incubated in a humidified incubator with 5% CO_2_ environment at 37°C for 1, 2, and 3 days. The cell viability was evaluated by MTT reduction assay. After desired days of incubation, the media from 96-well were suctioned out and treated with 200 μl of the MTT solution, by mixing the contents by side-tapping, and further on, these plates were incubated at 37°C for 2 h. After incubation, MTT solution was suctioned out and added with 200 μl of DMSO, which was subsequently rocked to form purplish blue-colored formazan solution. The solubilized formazan appearing from each well were transferred to fresh wells of 96-well plate for spectrophotometric analysis at 540 nm in an ELISA microplate reader (Molecular Devices, SpectraMax® Plus 384, Sunnyvale, CA, USA). The cell viability was obtained by comparing the absorbance of cells cultured on the nanofiber scaffolds to that of the control well containing DMSO. For cell checking attachment on nanofibers, the cells were allowed to grow for 3 and 12 days’ time, and media was changed after every 3 days. To check the cell morphology, cell fixation and dehydration was done by rinsing the samples twice with PBS followed by fixation with a 2.5 vol.% glutaraldehyde solution for 4 h. After cell fixation, the samples were rinsed with PBS and then dehydrated with graded concentrations of ethanol (20 vol.%, 30 vol.%, 40 vol.%, 50 vol.%, 70 vol.%, and 100 vol.% ethanol) for 10 min each. Finally, the samples were kept overnight in a vacuum oven and observed in FE-SEM to determine cell attachment. The samples for FE-SEM were coated by keeping the same conditions as described previously in the ‘Characterization’ section. However, the micrographs of each sample were taken at an accelerating voltage of 2 KV and with magnifications of 15 K.

## Results and discussions

**Figure 3:**
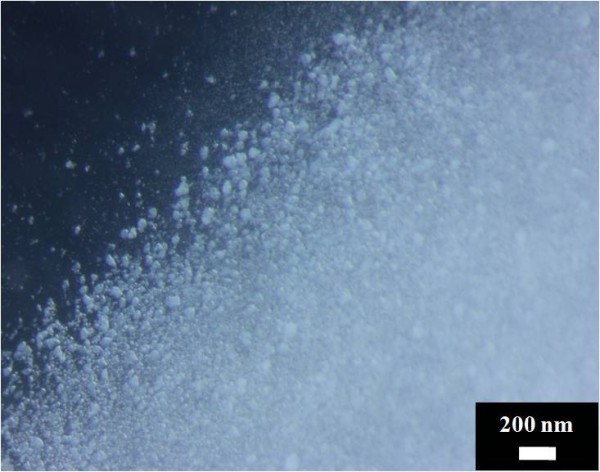
Optical micrograph of the composite solution containing silk/PEO and HAp/PEO after mixing using the threeway connector.

**Figure 4:**
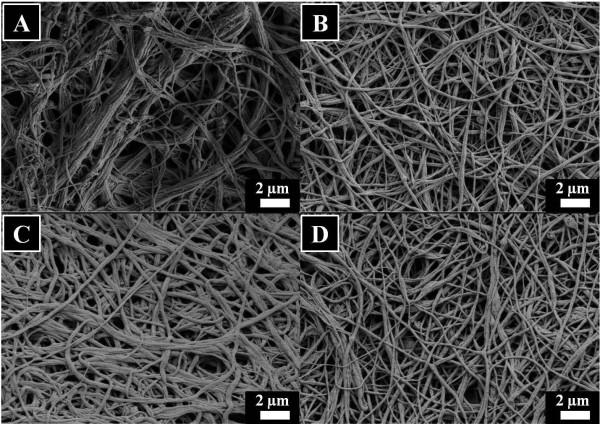
**Field emission scanning microscopy results.** Of the pristine silk fibroin nanofibers **(A)**, silk fibroin nanofibers modified with 10% HAp **(B)**, 30% HAp **(C)**, and 50% HAp **(D)**.

**Figure 5:**
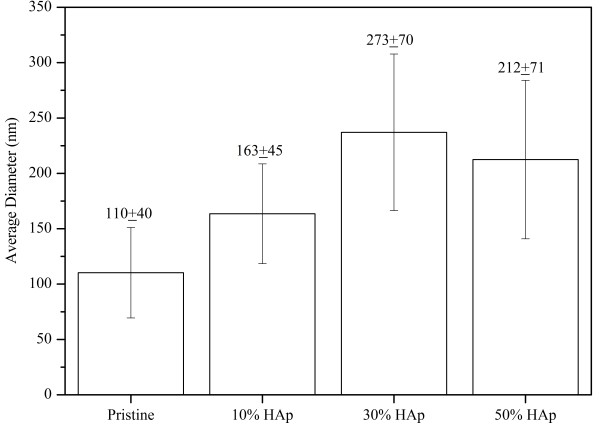
Average nanofiber diameters of the obtained nanofibers.

The three-way stopcock connector was used as the solution blending tool before ejecting the solution into nanofibers. In this regard, Figure 3 demonstrates the degree of dispersion of HAp NPs in the silk solution. This optical micrograph was taken from silk/PEO and HAp/PEO composite solution immediately after mixing using the threeway connector. In this figure, we can clearly observe that HAp NPs are completely dispersed in the silk solution, which further confirms that HAp NPs can be easily carried along with the electrospinning solution during fiber formation. Electrospinning of silk solutions containing various amounts of HAp NPs (i.e., 0%, 10%, 30%, and 50%) afforded in the fabrication of nanofibers with desirable morphology (Figure 4). Figure 4A represents the results after electrospinning of pure silk solutions; it can be observed that nanofibers are smooth, uniform, continuous, and bead-free. Moreover, its counterparts containing HAp NPs are represented in Figure 4B,C,D. By observing these figures, one can come up with a simple conclusion that general morphology had not been affected by the addition of HAp NPs. However, it can be observed that there is a reasonable increase in fiber diameters due to the addition of HAp NPs. To find out the actual effect caused due to the addition of HAp NPs on nanofiber, the average diameters of nanofibers were calculated from randomly selected individual fibers (100 diameters measured per sample) using the image analyzer software (Innerview 2.0). In this regard, Figure 5 presents the bar graphs for diameters calculated from each nanofiber combinations. It can be observed that pristine nanofibers had an average diameter of 110 ± 40 nm, and nanofibers modified with 10%, 30%, and 50% HAp NPs had increased diameters of 163 ± 45 nm, 273 ± 70 nm, and 212 ± 71 nm, which indicate the allocation of higher viscosity due to the presence of HAp NPs colloid which resulted in large droplet formation, giving it a tough bending instability during fiber formation and that finally resulted to the increase of the nanofiber diameters [26].

**Figure 6:**
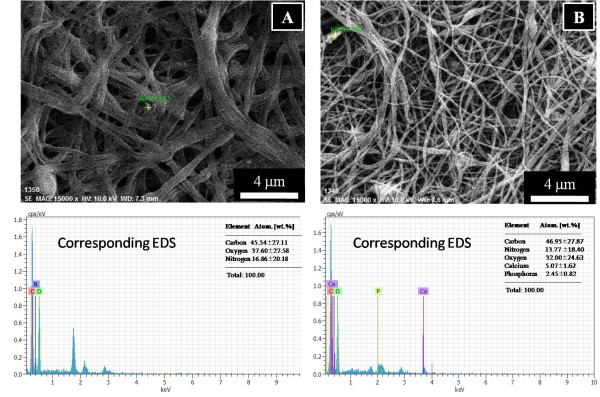
**Field emission scanning microscopy equipped with EDS results.** For the pristine silk fibroin nanofibers **(A)** and silk fibroin nanofibers modified with 10% HAp nanoparticles **(B)**.

To ensure that the added HAp particles are really present in/on nanofibers, FE-SEM equipped with EDS analysis was utilized for a comparative study of pristine and one of the modified nanofibers containing HAp NPs; the results are presented in Figure 6. Figure 6A shows the FE-SEM images, for pristine nanofibers indicating the point EDS taken at the center, and its corresponding EDS graph is presented underneath this figure. As shown in the inset (Figure 6A), weight percentage of pristine nanofibers contains (C, N, and O) elements only which symbolize the proteinaceous compounds originating from pristine nanofibers. Moreover, its counterpart (Figure 6B), the silk nanofibers incorporated with HAp NPs, shows the presence of (Ca and P) elements inside the nanofibers in addition of the other elements compared to that of the pristine one. The presence of these peaks clearly indicates the involvement of HAp NPs inside the nanofibers which were carried through designed electrospinning setup.

**Figure 7:**
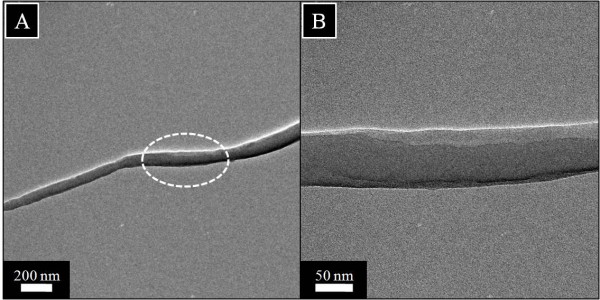
Transmission electron microscopy results of the pristine silk fibroin nanofibers in low (A) and high magnifications (B).

**Figure 8:**
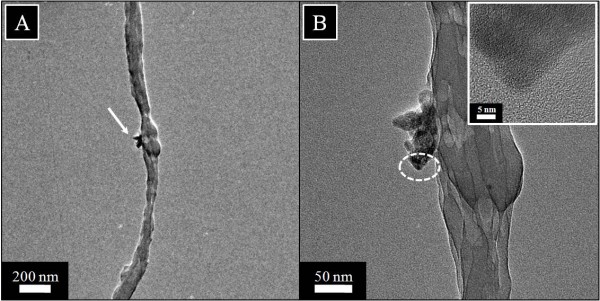
**Transmission electron microscopy results of silk fibroin nanofibers containing 10% HAp NPs in low (A) and high magnifications (B).** The inset in the figure **(B)** shows the HR-TEM of the encircled area.

Due to the poor resolution of scanning electron microscopy, it can only reveal the surface architect of materials, while internal contents often remain untracked. For this reason, we could not find the exact location of HAp NPs on nanofiber by FE-SEM. Therefore, we used TEM to investigate the location of HAp NPs inside the nanofibers. In this context, Figure 7A,B shows the TEM images in low and high magnifications, obtained after analyzing the pristine nanofibers, which are free of any NPs. In this figure, pristine nanofibers can be seen intact and/or aberrationfree, indicating its pristine nature. Moreover, the morphology of the nanofiber modified with HAp NPs shown in Figure 8B, for low and high magnifications, reveals clear appearance of HAp NPs in nanofibers. As indicated by an arrow (Figure 8A), we can see the separated HAp NPs at the centric position of the nanofiber. Moreover, in Figure 8B, the high magnification image of the marked area near HAp NPs on the nanofiber shows the inset figure indicating the HR-TEM of the encircled area. This inset in the figure shows apparent crystal patterns present to that of the HAp NPs in the nanofibers. Furthermore, these results clearly demonstrate the presence and location of HAp NPs in and around nanofibers.

**Figure 9:**
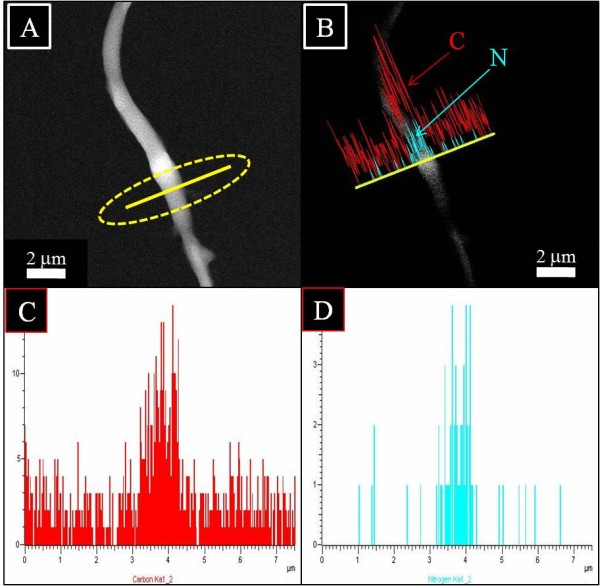
**TEM-EDS image of pristine nanofibers using silk/PEO solution.** Single selected fiber shows the area for line EDS **(A)**, the linear EDS analysis along the line appearing from nanofiber **(B)**, graphical results of line mapping for the compounds analyzed as carbon (red) **(C)** and nitrogen (cyan) **(D)**.

**Figure 10:**
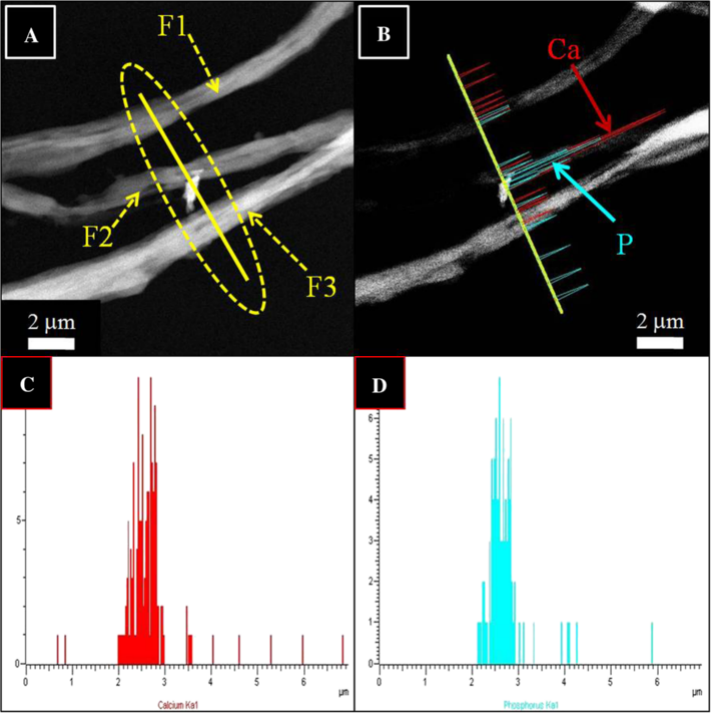
**TEM-EDS image of nanofibers prepared from a silk fibroin nanofiber modified by 10% HAp NPs.** Three fibers marked as F1, F2, and F3 selected for line EDS **(A)**, the linear EDS analysis along the line appearing from three nanofibers **(B)**, graphical results of line mapping for the compounds analyzed as calcium (red) **(C)** and phosphorous (cyan) **(D)**.

To investigate the location and chemical nature of HAp NPs over the nanofibers more precisely, TEM combined with the energy dispersive X-ray (EDS) analysis was done. Figure 9 shows the TEM-EDS results for pristine nanofibers. Figure 9A shows the single fiber under investigation, and the encircled area indicates line mapping. Figure 9B,C,D shows the spectra originating from the former figure (Figure 9A). In this figure, the spectra colored in red indicates carbon, and spectra in cyan indicates nitrogen, which further describes the chemical composition of silk fibroin used for electrospinning. In case of nanofibers modified with HAp NPs, Figure 9 shows the results of TEM-EDS. To get more insight about the location and chemical nature of nanofibers, areas near the site of investigation are encircled, and three fibers are coded as F1, F2, and F3. Two of them indicated as F1 and F3 appear as neat nanofibers without the presence of any extra structure (i.e., HAp), while the nanofiber which is centrally located in this figure shows poking out appearance of HAp within its alignment. Moreover, to get more clear confirmation with regard to the chemical compositions of each compound present in this selected area, Figure 10B,C,D shows the results of line mapping from the former figure (Figure 10A). In this figure, the encircled area near F1, F2, and F3 giving rise to different peaks in different colors are indicated. Briefly, main compounds have been identified as calcium (red) and phosphorous (cyan). From this figure, one can clearly reveal the presence of Ca and P that is more predominating from the central nanofiber (i.e., F2) region which further clarifies the presence of HAp NPs associated with modified nanofibers and simultaneously supports the simple TEM results (Figure 8).

**Figure 11:**
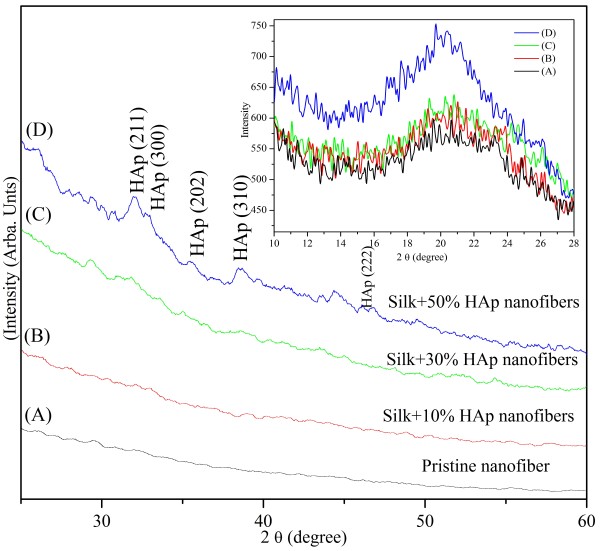
**The XRD results of the obtained nanofibers at 2*θ *values from 10° to 60°.** The inset in the figure shows the 2*θ* value from 10° to 28°. Pristine nanofibers (spectrum A), silk fibroin nanofibers modified with 10% HAp NPs (spectrum B), 30% HAp NPs (spectrum C), and 50% HAp NPs (spectrum D).

XRD can be utilized as a highly stable technique to investigate the crystalline nature of any material. Figure 11 shows the XRD data for the pristine silk nanofibers and its other modified counterparts facilitated using the stopcock connector to support the immediate mixing of aqueous silk/PEO solution and HAp/PEO colloids. In this figure, nanofibers modified with HAp NPs show various diffraction peaks (indicated by arrows) at 2*θ* values of 31.77°, 32.90°, 34.08°, 40.45°, and 46.71° that correspond to the crystal planes (211), (300), (202), (310), and (222), respectively, which are in proper agreement with the JCPDS database [27,28]. Unfortunately, the silk nanofibers modified with lower concentrations of HAp NPs did not exhibit the strong peak intensities as that of the higher ones showed. The rationale is that the hydroxyl and/or amide groups present in the silk fibroin can capture the calcium and phosphorous groups present in HAp NPs, thereby resulting in the covering of apatite nuclei to X-ray beams to be detected at lower concentrations. However, comparing the higher content counterparts obtained after the addition of HAp NPs, (i.e., silk + 50% HAp NPs) the spectra possess reasonably extra peaks located at the same diffraction angles as that mentioned in the JCPDS database [27,28]. Furthermore, the graph shows the spectra of nanofibers modified with lower concentrations of HAp NPs not showing strong intensity peaks than the higher concentrations. This may be the limitation with XRD technique or may be due to the masking of HAp crystals by silk fibroin. In order to understand the effect caused by the addition of HAp NPs on the nature of silk fibroin nanofibers and to simultaneously put more light on the crystallinity of silk fibroin in nanofibers, the inset in Figure 11 shows the diffraction peaks obtained at 2*θ* values from 10° to 28°. The broad diffraction peak in this inset shows the scatter peak with 2*θ* values of 21.9° which is indicating typical amorphous scattering pattern of amorphous silk [29]. Interestingly, it can be observed that this broad peak forms strong peak with increased intensity with nanofibers modified with HAp, which further indicates enrichment in the transformation from randomly arranged to crystalline βchain structure, in the case of nanofibers modified with HAp NPs.

**Figure 12:**
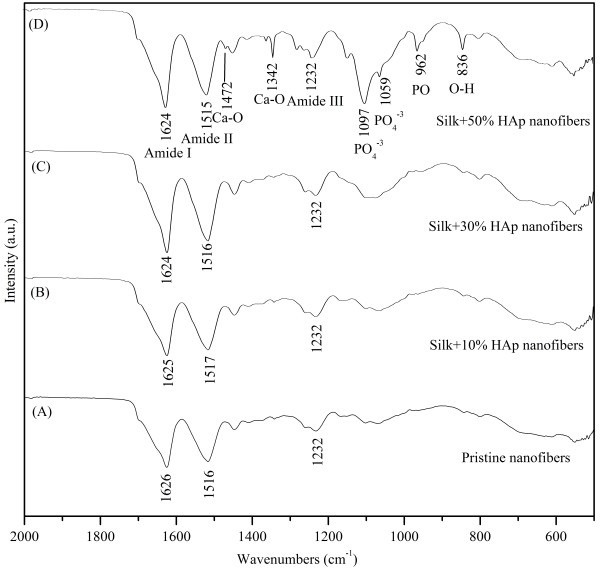
**The FT-IR spectra of the nanofibers obtained after electrospinning.** Pristine nanofibers **(**spectrum A**)**, silk fibroin nanofibers modified with 10% HAp NPs **(**spectrum B**)**, 30% HAp NPs **(**spectrum C**)**, and 50% HAp NPs **(**spectrum D**)**.

FT-IR can be used as an efficient tool to investigate the structural confirmations because of the knowledge of the vibration origins of the amide bonds, the sensitivity of some of these band positions to conformation, and the possibility of predicting band positions for a given helical or extended conformation [30]. The changes occurred on the band positions for pristine, and the one modified with HAp NPs is expressed in Figure 12. The vibrations occurred in pristine nanofiber due to amide Ι, amide II, and amide III bands can be seen at 1,626 cm^−1^, 1,516 cm^−1^, and 1,232 cm^−1^ which confirm the nature of the silk fibroin in the nanofibers. Moreover, nanofibers modified with HAp also showed the presence of these amide bands; however, there was a downshift of 1 to 2 units for amide Ι and amide II bands. The reason is to show that this shift can be attributed to conformational changes occurred in the silk fibroin from random coil structure to β-sheet confirmation due to the incorporation of HAp NPs [31,32]. The vibration modes caused due to the presence of Ca and P in nanofibers modified with HAp NPs can also be seen at different band positions. For instance, a small shoulder peaks at 1,472 cm^−1^, which indicates the existence of a Ca-O phase. The peaks appearing at 1,059 cm^−1^ and 1,097 cm^−1^ can be attributed due to the asymmetric stretching mode vibration in PO_4_^−3^, and a medium intensity band at about 962 cm^−1^ results from P-O asymmetric stretching of the stretching vibrations in PO_4_^−3^[33]. Also, a sharp peak at 836 cm^−1^ is assigned to the O-H bending deformation mode due to the presence of HAp NPs in the nanofibers. The intensity of these peaks increases as the amount of original HAp used to make colloidal solution for electrospinning increases.

**Figure 13:**
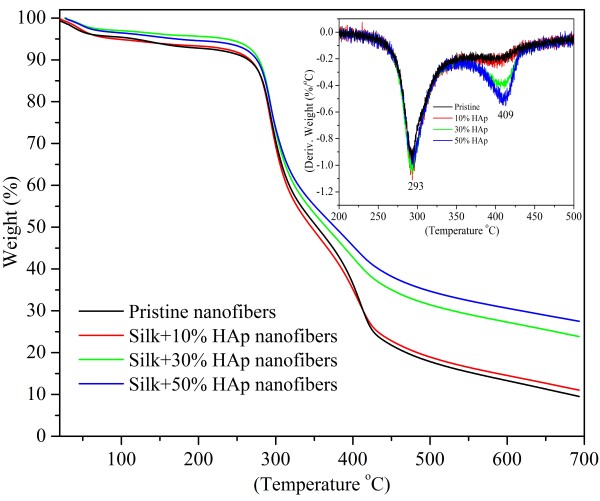
**The TGA results for the obtained nanofibers.** The inset in the figure shows the derivative of weight loss for the obtained nanofibers.

Figure 13 shows the results obtained after thermogravimetric analyses (TGA) of pristine and nanofibers modified HAp NPs. It was expected that the introduction of HAp NPs on the nanofibers would result in the improvement in thermal and crystalline properties of the nanofibers. After analyzing the data, it was observed that all the nanofiber samples showed initial weight loss of about 4% to 6% until 100°C, which is due to the removal of residual moisture. The onset temperatures of pristine nanofiber was calculated to be 269°C, and the nanofibers modified with HAp NPs represented higher onset temperatures of 273°C, 275°C, and 276°C. This high onset temperatures in case of nanofibers modified with HAp can be corroborated due to the β-sheet crystalline structures and covalent bonding of silk fibroin with HAp NPs, which result to the increase in the onset temperatures. The inset in the figure of the graph (Figure 13) represents the derivative of weight loss for nanofibers. As indicated in the inset in the figure, the first step degradation occurring in all nanofiber combinations can be clearly seen at 293°C which can be assigned due to the degradation of silk fibroins. Moreover, the nanofibers modified with HAp NPs show the second step degradation point at 409°C, which sharpens as the concentration of HAp is increased in nanofibers. Interestingly, it further clarifies that the molecular orientation and/or the crystallinity of silk fibroin can be improved by the incorporation of HAp NPs at higher amounts. At 693°C, the weight residues remaining for pristine nanofibers were calculated to be 9%, and the nanofibers modified by HAp NPs showed the increased residual weight remaining of 11%, 23%, and 27%. This increase in residual weights is due to the reason that HAp NPs had high thermal stability than the pure silk fibroin which probably helped the other modified counterparts to gain more residual weights of that of the pristine one.

**Figure 14:**
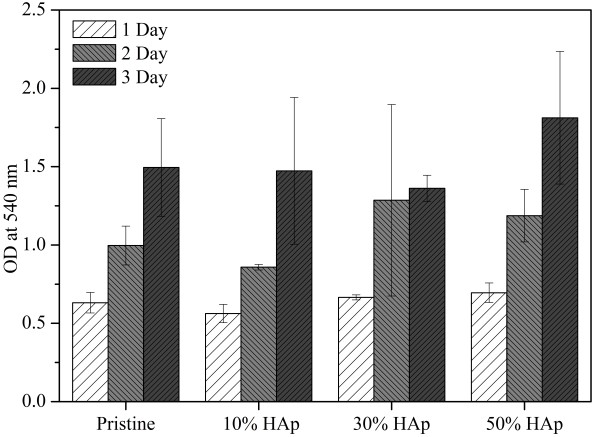
MTT assay results revealing cell viability after culturing the NIH 3 T3 fibroblasts in the presence of nanofibers.

Figure 14 represents the results obtained from MTT assay. In this figure, it can be observed that all the nanofiber combinations show the logarithmic phase of growth as the days of incubation pass (i.e., 1, 2, and 3 days). Moreover, the cell viability of nanofibers modified with HAp showed an increase in the growth as the concentration of HAp is increased. These results further suggest that used HAp NPs are non-toxic to cells, and there is a considerable positive impact induced by HAp NPs.

**Figure 15:**
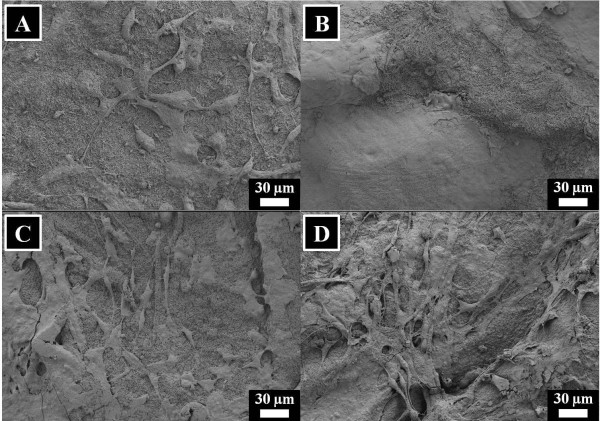
**Results of the cell attachment after culturing the NIH 3 T3 fibroblasts in the presence of nanofibers for 3 days.** For pristine silk fibroin nanofibers **(A)**, silk fibroin nanofibers modified with 10% HAp **(B)**, 30% HAp **(C)**, and 50% HAp **(D)**.

**Figure 16:**
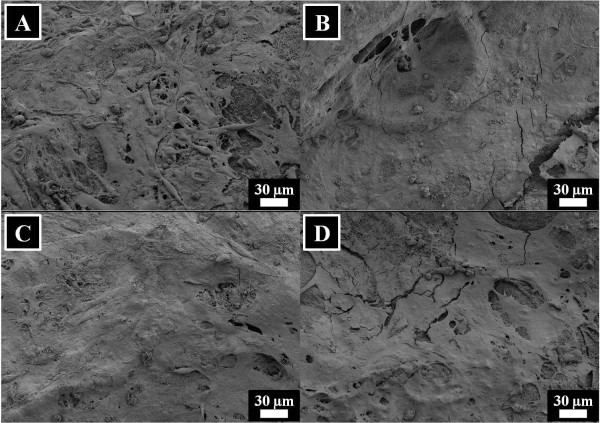
**Results of the cell attachment after culturing the NIH 3 T3 fibroblasts in the presence of nanofibers for 12 days.** For pristine silk fibroin nanofibers **(A)**, silk fibroin nanofibers modified with 10% HAp **(B)**, 30% HAp **(C)**, and 50% HAp **(D)**.

To find out the cell attachment on nanofibers, the results after culturing the fibroblast for 3 and 12 days is presented in Figures 15 and 16. In case of culturing the cells for 3 days, it can be seen that the cells are properly attaching on nanofiber surfaces. After looking on the cells, it is highly realized that the cells are stress-free and are growing in a healthy manner. Furthermore, the cell attachment results after culturing the cells for 12 days are presented in Figure 15. In this figure, we can see the confluent growth of cells on nanofiber surfaces which further indicates the non-toxic nature of nanocomposites. However, from these figures (i.e., Figures 15 and 16), it can be observed that cell attachment is independent to the presence of HAp in nanofibers.

## Conclusions

In conclusion, a highly trustable technique which employs the use of stopcock connector can be used to electrospun a blend solution of fibroin and HAp together in aqueous solutions, which is impossible if simple mixing procedure is followed. Without the use of any toxic chemical, this technique can yield nanofibers with desirable properties. The FE-SEM and TEM techniques can be used to figure out the location of HAp in nanofibers and simultaneously support the use of stopcock connector to electrospun silk fibroin and HAp NPs. Fourier transform infrared spectroscopy analysis indicated the chemical interaction occurring between HAp NPs and silk fibroin, which resulted in the transformation of random coil to β-sheet confirmation of silk fibroin. It can also be concluded that HAp NPs enhanced the β-sheet conformation of fibroin and resulted in the improvement of the properties of nanofibers. Generally, it is believed that the presence of highly favorable components likeHAp could improve the cell attachment; however, in our case, we find that the attachment of fibroblast on nanofibers is independent to the presence of HAp. Moreover, the cell viability of nanofibers can be improved by this technique.

## Competing interests

The authors declare that they have no competing interests.

## Authors’ contributions

FAS designed the study, carried out the experiments, and prepared the manuscript. HWJ, BMM, and HJP maintained the cell lines and provided vital information about the cell culture studies. OJL and JHK maintained the paperwork for obtaining the chemicals and arranging the facility to perform the characterization of materials. CHP supervised the whole work and attributed important part in the discussions of this manuscript. All authors read and approved the final manuscript.
